# Book Reviews

**Published:** 1900-09

**Authors:** 


TRANSACTIONS OF THE AMERICAN ASSOCIATION OF OBSTETRICIANS AND GYNECOLOGISTS. Volume XII. For the Year 1899. Edited by William Warren Potter, M. D., Secretary. Philadelphia: W. J. Dornan, Printer. 1900.

This volume contains the proceedings of the association during its twelfth annual meeting, held at Indianapolis, Ind., September 19-21, 1899, under the presidency of Dr. Edward J. Ill., of Newark, N. J. The president chose for the subject of his address, "The rights of the unborn; the prevention of conception." He presented concisely and ably the most enlightened and conservative view of the perplexing problems comprehended by the title.

One of the prominent features of the meetings of this association is the holding of a session for the presentation of specimens and discussions as to their clinical and pathological significance. These specimens are photographed and reproduced in half-tones in the book. This most excellent method serves to stimulate pathologic research and to lead to the proper interpretation of pathological lesions. It brings into closer relation clinical experience and pathologic facts.

There are thirty-three contributions in the volume, many of which are superbly presented and admirably discussed, while all are of interest and more or less instructive.

We cannot speak of them in detail and do not wish to make invidious distinction, nevertheless, we feel impelled to mention the article of Dr. John W. Ballantyne, of Edinburgh, on "The antenatal factor in gynecology," as a contribution to the general knowledge of a subject that has only a sparse literature.

The memorial address upon the life and character of Lawson Tait, by Charles A. L. Reed, of Cincinnati, which is the final article in the book, is a masterful presentation of the life-history of one of the great lights of modern medicine, who has been a distinctive contributor to gynecological science. Dr. Reed's marvelously eloquent address will be quoted ages hence whenever a model is desired or an exemplar pointed out.

ON DIABETES MELLITUS AND GLYCOSURIA. By EMIL KLEEN, Ph. D., M. D' Octavo, 313 pages. Philadelphia: P. Blakiston's Son & Co., 1012 Walnut Street. 1900. [Price, cloth, $2.50 net.]

The syndrome, diabetes mellitus, of which the chief and most constant symptom is glycosuria, has long been the subject of study by pathologists and clinicians. Not yet, however, has a satisfactory conclusion been reached as to its causes, true pathology, or best treatment. This contribution of Kleen will aid very materially in the solution of many doubtful points connected with diabetes.

The author has had exceptional opportunities to pursue his investigations and a unique experience in dealing with the disease. His residence at Carlsbad has served to develop and mature both his studies and experience. It is, so to speak, a storm center of diabetes. Being a disease of the wealthy and sedentary classes, diabetics in great numbers frequent the German spas and especially Carlsbad. The habitually luxurious liver is especially prone to the disease; whereas, the working man is astonishingly exempt from it.

Slight glycosurias play an interesting and important part in the economy, and bear an intimate relation to other maladies. True, so-called functional glycosuria may be comparatively unimportant, per se, but it may serve to explain obscure symptoms and lead to accuracy in diagnosticating other and even graver conditions.

Kleen deals with the whole subject with intelligence, points out its direct and remote influence on the economy, discusses symptoms and complications of mild and severe forms of the disease with clearness, and discourses upon treatment in an intelligent manner. The mortality among children of diabetic parents is high, hence the importance of preventing proposed marriages between confirmed diabetics or even between a diabetic on the one side and a healthful partner on the other. Prophylactic measures against diabetes of rigid nature are often needed and Kleen points out the fact as well as the method of carrying out the needed measures.

In the treatment Kleen places less reliance upon drugs than upon hygiene and dietetics. He, however, does not undervalue well-directed drug treatment as an adjunct to the other measures recommended. He makes practical application of the most approved medicines with a scientific discretion.

This is a treatise that will prove helpful to the general practitioner who will appreciate its value as a clinical guide. It deserves the approbation of the profession of medicine.

Normal Histology. By Edward K. Dunham, M. D., Professor of General Pathology, Bacteriology and Hygiene in the University and Bellevue Hospital Medical College, New York. New (2d) edition. In one very handsome 8vo volume of 319 pages, with 244 illustrations. Philadelphia and New York: Lea Brothers & Co., Publishers. 1900. [Price, cloth, $2.50 net.]

The first edition of Dunham's histology was reviewed in the April, 1899, number of this journal, page 717, and the opinion expressed that it would prove highly successful, because the author covered the enlarge, rearrange and revise, and so make this volume as complete and accurate as the present state of scientific medicine will permit.

The binding, paper and print conforms with other numbers of this series. W. C. K.

---

MANUEL COMPLET DE GYNÉCOLOGIE MÉDICALE ET CHIRURGICALE. Par A. LUTAUD, Professeur de Gynécologie, Médecin Adjoint de Saint Legare, etc., etc. Nouvelle édition entièrement refondue. Contenant la technique opératoire complèt, pp. 712, et 607 figures dans le texte. Paris; A. Maloine, Editeur, 23–25 rue de l'École de Médecine. 1900. From Mariani & Co., New York.

This well known author presents to the profession a new edition of a treatise that has taken an established place in the literature of gynecology. It gives in minute detail all the minor gynecological procedures as well as all major operations falling within the domain of gynecological surgery.

Professor Lutaud is to be classed as a practical gynecologist, who is not devoted to the exploitation of any particular fad, but gives the best instruction on all accepted measures that have in view ultimate cure of the patient.

The illustrations are to be commended with special insistence; they are so clear, so well drawn, and withal so copious, that a study of them will enable even a novice to do many operations well that he may not have witnessed. The work before us is in the original French, hence possesses an additional charm to students of that language.

